# Assessment of Microbial Transmission by Insect Vectors in Healthcare Settings: A Systematic Review

**DOI:** 10.1017/ash.2025.361

**Published:** 2025-09-24

**Authors:** Rishi Patel, Barrett Meeks, Nicholas Schouten, Jennifer Hanrahan, Daniel Stuart, Kendra Rumbaugh, Stephen Diggle, Stephanie Stroever

**Affiliations:** 1 Texas Tech University Health Sciences Center; 2 Eastern Virginia Medical School; 3 Georgia Tech

## Abstract

**Background:** Multi-drug resistance is increasing, and insects may serve as critical reservoirs for these bacteria. While insects are part of the world’s natural flora and exist even in healthcare settings, their presence is often overlooked or disregarded. Current healthcare safety guidelines focus on keeping insects out but fail to address their potential contribution to nosocomial pathogen transmission, This study aims to determine the extent of knowledge regarding microbial transmission by insects in a healthcare setting. **Methods:** The systematic literature search utilized subject headings and preferred index terms such as “insects,” “microbial transmissions,” “insect vector,” “insecta,” and “cross infection” to construct a reproducible MEDLINE search within PubMed. This search was translated across Embase, CENTRAL, and select supplemental resources. Retrieved studies exceeded 1000 records, which were deduplicated using Covidence software, leaving 655 eligible articles for screening. A total of 36 peer-reviewed studies documenting insects in healthcare settings (e.g., hospitals, clinics, rehabilitation centers, ambulatory centers) were included and underwent data extraction and analysis. Excluded studies were non-English without translation or irrelevant to healthcare. Outcomes included identification of colonizing organisms, evidence of microbial transmission, and prevalence of multidrug-resistant pathogens carried by insects. Risk of bias was evaluated using the LEGEND criteria, and extracted data were synthesized following the Cochrane Review for Systematic Reviews in conjunction with the Joanna Briggs Institute (JBI) frameworks. **Results:** Of the analyzed studies the majority were from low- to middle-income countries and included the study of common insects such as cockroaches, ants, and various classes of flies. Studies included the examination of external surfaces and internal contents of the insects via evidence-based, aseptic collection and laboratory processes. Few studies demonstrated the likely transmission of bacteria via insect vector, though all studies demonstrated robust colonization of insect vectors with pathogenic microbes including multi-drug resistant bacteria. **Conclusion:** This review highlights the potential role of insects in microbial transmission in healthcare settings. However, most studies failed to establish a direct link between insects and pathogen transmission. Further research into areas such as multi-drug resistance transmission via insect vectors is needed.

